# The effects of athletes’ moral identity on clean sport behavior intentions: a cross-national study of the mediating role of moral disengagement and self-efficacy

**DOI:** 10.3389/fspor.2026.1791964

**Published:** 2026-03-26

**Authors:** Martina Giorgi, Andrea Chirico, Tommaso Palombi, Vindice Deplano, Daniel Gotti, Alessandra De Maria, Federica Galli, Luca Mallia, Roberto Codella, Arnaldo Zelli, Fabio Lucidi

**Affiliations:** 1 Department of Psychology of Social and Developmental Processes, Sapienza University of Rome, Rome, Italy; 2 Department of Biomedical Sciences for Health, University of Milan, Milan, Italy; 3 Department of Movement, Human and Health Sciences, University of Rome “Foro Italico”, Rome, Italy

**Keywords:** clean sport behavior intentions, cross-national study, doping prevention, moral disengagement, moral identity, quantitative study, self-regulatory efficacy, socio-cognitive model

## Abstract

**Introduction:**

In recent years, doping has increasingly been recognized not just as a health risk for athletes, but also as a factor undermining the credibility of sports as a whole. The scientific discussion is gradually shifting: rather than simply predicting risky behavior, there is growing emphasis on fostering a genuine “clean sport identity,” meaning a conscious commitment to shared ethical values. In this study, we draw on both Social Cognitive Theory and moral identity perspectives to explore which socio-cognitive variables may influence the intention to support ethical sporting behavior.

**Methods:**

A total of 429 track and field athletes from Italy (*n* = 229) and France (*n* = 200) participated in the study. Participants completed validated questionnaires measuring moral identity, moral disengagement, self-regulatory efficacy, and clean sport behavior intentions. Structural equation modeling and multigroup invariance analyses were employed to test the theoretical model and examine cross-national differences.

**Results:**

The results demonstrate that moral identity is negatively associated with moral disengagement and positively associated with self-regulatory efficacy. Moral disengagement was identified as the strongest predictor of clean sports behavior intentions. Mediation analysis indicated that moral identity influences clean sports behavior intentions primarily by inhibiting mechanisms of moral disengagement. When comparing groups, we observed notable cross-national differences. Specifically, the link between moral identity and moral disengagement appeared stronger among French athletes, whereas self-regulatory efficacy had a more pronounced impact on Italian athletes’ intentions to engage in clean sports behavior.

**Discussion:**

These results underscore the importance of incorporating value-driven approaches and fostering moral identity within anti-doping interventions. Preventive strategies should focus on minimizing cognitive rationalizations for unethical conduct and strengthening athletes’ ability to self-regulate when facing external pressures, ultimately supporting a long-term dedication to clean sport.

## 1 Introduction

The report on anti-doping rule violations (ADRVs) shows that out of a total of 149,758 samples collected by ADOs in the years leading up to 2020 and subsequently analysed by WADA-accredited laboratories, 1,007 samples (0.67%) were reported as adverse analytical findings (AAF). Of these, 672 samples (66%) were found to be ADRVs (resulting in sanctions). Of these, athletics is one of the sports with the highest number of ADRVs committed by athletes, with 107 cases (1).

In recent decades, several factors have influenced the trend of improving physical performance in sports. From a contextual perspective, the commercialization of sports (2, 3) and the inherent escalation of competitiveness at both amateur and elite levels deserve particular attention [see (2, 4)]. Athletes are frequently compared with the pressures and expectations tied to their performance, factors that can shape their growth and achievements in the sporting domain (3, 5). These ongoing challenges and demands may, in turn, influence, either directly or indirectly, whether athletes choose to uphold ethical standards or resort to doping.

The World Anti-Doping Agency (1) defines doping as the use of prohibited substances or methods by athletes to artificially enhance their physical or mental performance during competitions or training sessions. This definition includes the use of drugs and non-pharmacological interventions that provide an unfair advantage, compromise the athlete's health, and violate the principles of sports ethics. The use of prohibited substances or methods has significant implications for health (6–8), psychological well-being, and the ethical development of young athletes [(9); cfr (6)]. Understanding this complex mechanism is crucial for addressing the risks associated with the use of prohibited substances or methods and implementing training intervention programs that target behavioral variables related to athletes' conduct [cf. (6)].

Goal-oriented theory, which deals with the determinants of human behavior, typically defines it as an intentional process driven by the identification, planning, and achievement of specific goals. This theoretical model, such as the Theory of Planned Behavior (10) of Socio Cognitive Theory (11), emphasizes the importance of individual attitudes, expectations, and contexts in shaping the actions and choices of individuals. What distinguishes these theories is that behavior, rather than being random, results from structured cognitive, affective, and decision-making processes that include anticipating outcomes and evaluating one's capabilities (12, 13). Such approaches are commonly used to explain and predict complex behaviors, also in sports contexts (14). Bandura's (11) SCT has been adopted in several studies to understand the complexity of phenomena involved in the psychology of doping (15). The psychological model explains human learning and behavior as a reciprocal interaction between personal, environmental, and behavioral factors. The decision to use doping substances, like other forms of illicit behavior, can depend on an individual's personal resources and self-regulatory abilities, which are part of the concept of human agency in behavioral phenomena (16).

Among the variables outlined in SCT, self-efficacy is the most related to human agency. Introduced by Bandura (11), self-efficacy refers to an individual's belief in their ability to pursue specific goals, exercise control over their actions, and direct their behavior towards purposeful choices. Self-efficacy is not just an assessment of one's competence but a dynamic construct that profoundly impacts motivation and commitment in challenging situations. Bandura emphasizes that this perception is influenced by factors such as direct experiences of success, observation of others, social persuasion, and managing emotional states. On the same side, self-regulatory efficacy pertains to the perceived capacity to govern one's thoughts, emotions, and behaviors in pursuit of long-term goals, particularly when faced with obstacles, distractions, or temptations. In essence, it embodies the belief in one's ability to manage oneself, such as exercising self-control, persisting through challenges, and monitoring progress, rather than simply adhering to a set task [cfr (11, 16)]. It is crucial in regulating human behavior and shaping decision-making (11, 16), particularly in the context of doping (17). Specifically, in the context of doping, it is particularly relevant in resisting external pressures that are prevalent in competitive environments, such as sports (14).

The human decision process is linked to moral aspects when considering illicit behavior. In the SCT, the concept of moral disengagement also involves the use of prohibited substances or methods, as proposed by Bandura (16, 18), which highlights cognitive mechanisms that enable individuals to justify behaviors that violate their ethical or moral principles. Individuals may minimize the consequences of their actions, diffuse responsibility, or attribute blame to others to alleviate guilt and engage in behaviors they would otherwise consider unacceptable (19).

Regarding morality, Aquino and Reed (20) developed a moral identity model grounded in social cognitive theory to explain moral behavior. Moral identity is a psychological construct that self-regulates moral behavior (21, 22). According to Aquino and Reed (20), it represents an understanding of the self that is organized around moral traits. This conception is shaped by mental frameworks relating to social contexts, such as groups, abstract ideals, or known and unknown figures [cfr (20)]. In sports, moral identity is closely associated with ethical values. The WADA Code (updated in 2021) emphasizes that “anti-doping programs aim to preserve what is inherently valuable in sport.” This intrinsic value is defined as “the spirit of sport,” which encompasses values such as ethics, fairness, honesty, health, performance excellence, character and education, enjoyment, teamwork, dedication, commitment, respect for rules and laws, respect for oneself and fellow participants, courage, community, and solidarity (23). These values are closely tied to the concept of moral identity (24). Athletes who embrace these principles cultivate a distinct identity that aligns with their values, making them less inclined to resort to performance-enhancing substances or illicit methods (3, 25).

Relatedly, research on doping has highlighted the critical role of socio-cognitive factors in understanding the use of prohibited substances or methods among athletes, such as, for instance, moral disengagement mechanisms, that is, one's tendency to mentally “disengage” from the moral consequences of one's action. Bandura (16) conceptualizes moral disengagement as a process through which individuals selectively deactivate their internal moral controls, in particularthe sense of guilt. These personal mechanisms enable the deflection of moral self-sanctions, leading to various forms of misconduct. Such strategies can take multiple forms: harmful behavior may be deemed acceptable if it is perceived to serve socially beneficial or moral purposes (moral justification); it may be downplayed or obscured through language (euphemism and twisted language); it can be compared to more egregious acts (exonerative comparison); personal accountability may be evaded (displacement and diffusion of responsibility); Bandura's (16) conceptualization of moral disengagement describes the process through which individuals selectively suspend their internal moral constraints. This mechanism facilitates the circumvention of moral self-sanctions, thereby paving the way for engagement in diverse unethical behaviors. These strategies manifest in several key ways: harmful conduct can be re-framed as serving a more significant, socially beneficial, or moral goal (moral justification); the severity of the transgression can be obscured or downplayed through linguistic manipulation (euphemism and twisted language); the action can be made to appear less problematic by contrasting it with more egregious acts (exonerative comparison); or personal culpability can be evaded (displacement and diffusion of responsibility); the social consequences of one's actions may be minimized, ignored, or distorted (misrepresentation of harm); responsibility may be assigned to others or to the victim (ascription of blame); or attempts may be made to strip the victim of their human qualities (dehumanization), all these have been declined also in sports context (14).

A meta-analysis by Ntoumanis et al. (15), evaluating the most critical predictors of doping, found that athletes with higher self-efficacy in resisting temptation were less likely to use Prohibited substances or methods. In turn, those with high levels of moral disengagement were also more prone to using these substances. Lucidi and colleagues (2008) investigated the role of moral disengagement in young athletes, revealing that they often justify the use of doping substances through cognitive mechanisms, including moral disengagement. In contrast, increased self-confidence in resisting social pressure is an essential protective factor [(26); cfr (14)].

Moral identity has also been identified as a deterrent against the use of prohibited substances or methods. Indeed, some studies confirm an inverse relationship between moral identity and the propensity to dope, indicating that athletes with a strong moral identity are less likely to consider using Prohibited substances or methods (27, 28). Furthermore, Ring and colleagues (28) discovered a positive correlation between moral identity and self-regulatory efficacy, suggesting that athletes with a strong moral identity tend to be more confident in resisting the temptation to use Prohibited substances or methods.

The growing emphasis on moral identity as a key factor in the use of prohibited substances or methods has led to a shift in the anti-doping literature towards prevention strategies that foster ethical and doping-free sports behavior. A central concept that has emerged from this mainstream is the idea of a “clean sport identity,” which is essential to these efforts (29). The concept of clean sport identity can be understood as a multidimensional framework that weaves together moral principles, behavioral intentions, and the shared social significance of being recognized as a “clean” athlete [see (4, 30–32)]. Within this framework, clean sport is viewed as extending beyond the simple rejection of doping; it embodies a more comprehensive self-concept rooted in fairness, integrity, and the congruence between personal values and athletic behavior (33). This perspective brings together individual moral dispositions, overt actions, and the shaping role of the social environment, such as coaches, teammates, and other influential figures (34, 35). Although the clean sport identity model underscores the interconnectedness of moral, behavioral, and contextual factors, it also acknowledges the persistent influence of socio-cognitive processes. Instead, it suggests that mechanisms such as moral disengagement and self-regulatory efficacy may be necessary, but are not sufficient alone to explain sustained commitment to clean sport (36, 37).

In the present study, clean sport identity is not operationalized as a unified, validated construct. Instead, a theoretically integrated model is examined, incorporating a value-based dimension (moral identity), core socio-cognitive processes (moral disengagement and self-regulatory efficacy), and a proximal behavioural dimension represented by clean sport behavioral intentions. In this context, behavioral intentions are considered an outcome reflecting athletes' orientation toward acting consistently with clean sport values (38). In fact, new frameworks stress the need to capture not just the lack of doping, but also athletes' active commitment to clean sport (38). Socio-cognitive theories see behavioural intentions as the closest predictor of action and a solid indicator of behavioural orientation. Measuring clean sport behavior intentions helps show athletes' commitment to clean sport values, without confusing intention with identity.

Recent literature on doping has attempted to integrate these theories and variables into cohesive models. For example, notably, the research conducted by Kavussanu and colleagues (39) reveals that behavioral intentions are influenced by a range of moral psychological variables at the individual level, such as moral identity, moral disengagement, and feelings of guilt, as well as contextual factors like the moral atmosphere. Similarly, the study conducted by Stanger and Backhouse (40) demonstrates that moral disengagement is a predictor of doping behaviors, while moral identity has a negative correlation with doping. However, at the state, none of the recent literature addresses a complete model of the clean sport identity testing relationship between values, socio-cognitive aspects (e.g., self-regulatory efficacy, and moral disengagement) in predicting clean sports behavior.

## 2 Materials and methods

### 2.1 Present study

**Figure 1:**
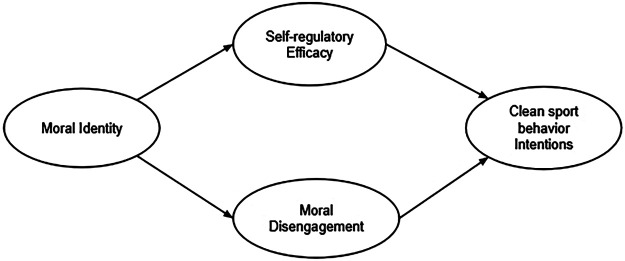
Hypothesized theoretical model.

The study is part of an interventional anti-doping project funded by WADA, involving Italian and French athletes. The project aims to evaluate intervention programs to foster clan sports identity in Italy and France. The present research study aimed to examine the socio-cognitive variables associated with clean behaviors (e.g., intention to act ethically) through SEM analysis, to develop intervention programs. The hypothesized model is represented in Figure 1. The study also aimed to validate measures through cross-national invariance analysis of the key variables in two samples of Italian and French athletes.

### 2.2 Participants

The study involved 501 (294) Italian and (207) French track and field athletes. Participants were recruited through federation networks, which may have introduced selection bias toward athletes already engaged in structured sport environments and potentially exposed to similar ethical education.

Of the 501 questionnaires that were returned, 72 were omitted from the analysis because they were not completed. The data eligibility refers to 429 sample athletes from Italy (*n* = 229) and France (*n* = 200). The sample used in this study has not been used in previous publications or analyses. This dataset is original to the current study.

### 2.3 Procedure

In 2024, a booklet of questionnaires was originally administered to 229 young Italian and 200 French track and field athletes practicing at amateur or competitive levels. Participants were recruited using a direct network from National Athletic Federations and Associations and asked to fill out the questionnaires through the online software “Qualtrics” (https://www.qualtrics.com). The study's general purpose and participants' rights to anonymity and confidentiality were made clear to all participants. The survey took approximately 20 min to complete. The survey was administered in Italian and then translated into French with a back-translation procedure (41).

### 2.4 Ethical aspect

The Ethics Committee at “Sapienza” University of Rome has approved the study protocol (research ID: 363/2025 rev. 1 dated 22/09/2025). All participants received information about the study and signed an informed consent. The patients/participants [legal guardian/next of kin] provided written informed consent to participate in this study.

### 2.5 Measures

Sports values were assessed through a modified version of the Moral Identity Scale (20), using the values defined by WADA in the “Spirit of Sport” (30). The scale in our adaptation presents a stem “Please read the following adjectives used to describe a person:” the scale presented the values presented by WADA Spirit of Sports (e.g., health-consciousness); to the participants were asked to answer to a 5-items 7-point Likert scale ranging from “completely disagree” to “completely agree”. Examples of the item were: “It would make me feel good to be a person with these characteristics.”

Moral disengagement mechanisms related to doping substance consumption were assessed through the Doping Moral Disengagement (14), a 4-items scale on a 5-point Likert scale ranging from “completely disagree” to “completely agree”. Examples of the item were: “Compared to the harm caused by alcohol and tobacco, using banned substances in sport is not that dangerous.”

Self-regulatory efficacy related to athletes' perceived ability to stick with their own values in sports contexts was assessed through a Doping Self-Efficacy Scale (DSES) (14), a 4-item scale referring to situations involving the use of prohibited substances in sport. Participants were asked to indicate how capable they feel of avoiding prohibited substances to enhance their sports performance, on a Likert scale ranging from “not at all capable” to “completely capable”. Examples of items were: “How capable do you feel of avoiding the use of prohibited substances when most athletes in your sport use them.”

Clean-sports behavior intentions were assessed through a 3-items scenario inventory, using a hypothetical scenario simulating real situations in which athletes might consider using prohibited substances (30, 42). Although the original measure by Kavussanu et al. (42) assessed doping likelihood (i.e., the probability of engaging in doping behavior), it has been adapted in its response format. The structure of the scenario closely follows previous clean sport research employing high-stakes hypothetical situations with no detection risk [e.g., (30, 38)].

Participants were presented with the following scenario describing a high-pressure competitive context in which a prohibited substance could enhance performance without risk of detection: “It is the week before the most important competition of the season. Recently, your performance has fallen below your best. You don't feel you are in the necessary physical shape for this competition, and you are worried about how you will perform. You mention this to a teammate, who tells you that they are using a new substance that has improved their physical condition and performance. The substance is banned in sport, but there is no chance of you being caught”. Following the scenario, participants responded to 3 items assessing their likelihood of promote clean sport behavior intentions [e.g., “If you were in this situation, how probable is it that you would compete clean (i.e., drug free)?”]. Responses were provided on a 7-point scale (1 = not at all likely; 7 = very likely). The full wording of the scenario and all items is reported in Supplementary Material 1.

### 2.6 Data analysis

For the data analysis, RStudio software has been used (RStudio version 3.6). R packages used are SemPlot, SemTools, and Lavaan (43). The analysis of data proceeded in the following steps.

Reliability of the tools used in the survey, to determine the internal reliability of the scales, Cronbach's *α* and omega, McDonalds were calculated for each scale or subscale for both countries: Italy and France.

Correlation analysis, a bivariate correlation analysis was conducted to examine the relationships between the key variables of the study.

Factorial structure and invariance analysis, confirmatory factorial analysis, and Invariance analysis were conducted to test whether the structure of each measurement was invariant between Italian and French athletes. Four levels of invariance were tested: configural, metric, scale, and strict.

A multigroup SEM analysis of the hypothesized model linking the key latent variables was first tested on the whole sample. Subsequently, we tested the relationships linking the model's variables to assess differences between countries. The hypothesized model considered moral identity as the predictor that influenced clean sport behavior intentions through the mediation of moral disengagement and self-regulatory efficacy.

## 3 Results

### 3.1 Descriptive analysis and reliability

The normality of the distribution has been assessed using Skewness and Kurtosis, with all variables falling within the range of −1 to +1. The Italian sample consists of 51.06% males, 47.52% females, and 1.41% identifying as non-binary or preferring not to say, with a mean age of 19.56 years (SD = 4.18). In contrast, the French sample includes 57.73% males, 39.69% females, and 2.58% identifying as non-binary or preferring not to say, with a mean age of 29.32 years (SD = 14.35).

Regarding the level of athletic involvement, the Italian sample features 23.08% of athletes participating at the amateur level, 8.41% at the provincial level, 11.78% at the regional level, 9.38% at the national level, and 1.20% at the international level (mean = 2.2, SD = 1.22). In comparison, the French sample comprises 7.45% of athletes at the amateur level, 3.13% at the provincial level, 12.02% at the regional level, 19.23% at the national level, and 4.33% at the international level (mean = 3.21, SD = 1.21).

Results of the reliability analysis showed an acceptable level of reliability for each scale, with Cronbach' *ɑ* varying from.689 to.974 and a McDonald' *ω* varying from.713 to.974 (see Table 1 for details).

**Table 1:** Correlation and reliability of all the scales.

Variables	Moral disengagement	Moral identity	Self-regulatory efficacy	Clean sport behavior intentions	*α*	*ω*
Moral disengagement	—				.689	.713
Moral identity	−0.275***	—			.856	.859
Self-regulatory efficacy	−0.200***	0.105*	—		.974	.974
Clean sport behavior intentions	−0.465***	0.127**	0.186***	—	.877	.887

Cronbach's Alpha (*α*), McDonald Omega (*ω*).

* *p* < .05.

** *p* < .01.

*** *p* < .001.

### 3.2 Correlation

The results of the correlation analysis which was run to test the linkages among the socio-cognitive variables included in the study (Moral Disengagement, Moral Identity, Self-regulatory efficacy and Clean sport behavior intentions) are shown in Table 1. Overall, there were strong associations among the key variables and these associations were in the expected directions.

### 3.3 Invariance analysis

In order to test the construct validity and the invariance of the measures of the study, invariance analysis was conducted by testing the structure of the model factor for each measure, considering a *Δ*CFI of 0.01 as the cut-off (44). Considering the overall model and taking into account the parsimony criterion, given higer RMSEA major.08, following modification index, item 4 of Doping Moral Disengagement scale was eliminated, item 4 of Moral Identity scale was eliminated and items 3, 5 of DSES scale were eliminated.

Analyses of measurement invariance revealed that the underlying factor structures were comparable for both the Italian and French samples. All constructs demonstrated metric invariance, indicating that factor loadings were equivalent across the two groups.

Scalar invariance was confirmed for moral identity and self-regulatory efficacy, but not for moral disengagement, pointing to only partial equivalence of item intercepts between the groups. Notably, strict invariance was established for moral identity and self-regulatory efficacy, while moral disengagement fell short of this criterion, implying that residual variances differed across samples (refer to Table 2 for model fit statistics, *Δ*CFI, and *Δ*RMSEA values). Overall, these findings indicate that the measures demonstrate adequate cross-national comparability, supporting subsequent multigroup SEM analyses.

**Table 2:** Invariance analysis of moral disengagement, moral identity, self-regulatory efficacy.

Construct	Measure	*χ* ^2^	RMSEA	*Δ*RMSEA	CFI	*Δ*CFI
Moral disengagement	Configural	24.977	.084		.934	
	Metric	31.380	.077	−.007	.928	−.005
	Scalar	47.489	.088	.011	.871	−.057
	Strict	148.870	.162	.073	.473	−.397
Moral identity	Configural	9.591	.082		.995	
	Metric	15.413	.076	−.005	.998	.003
	Scalar	16.671	.056	−.019	.998	−.000
	Strict	33.185	.081	.024	.996	−.001
Self-regulatory efficacy	Configural	9.282	.079		.998	
	Metric	21.980	.101	.021	.995	−.002
	Scalar	30.812	.100	−.001	.992	−.003
	Strict	54.378	.117	.017	.989	−.003

### 3.4 Structural equation modeling

#### 3.4.1 Measurement model

A measurement model was specified to examine the factorial validity of the adapted clean sport behavior intention scale. The scale consisted of three scenario-based items that assessed athletes' likelihood of promoting clean sport behavior intentions in a high-pressure competitive context. Responses were recorded on a 7-point scale, and items were reverse-coded so that higher scores reflect stronger clean sport behavior intentions.

A one-factor confirmatory model was estimated. Consistent with expectations for a single latent construct, all three items loaded strongly on the latent factor, with standardized loadings of .86, .79, and .87, respectively. These loadings indicate that each item contributed substantially to the measurement of clean sport behavior intentions and support the unidimensional structure of the scale.

Internal consistency analyses further supported the reliability of the adapted measure (details are reported as Supplementary Material 2). Taken together, the strong factor loadings, conceptual coherence of the scenario-based items, and internal consistency evidence support the factorial validity of the three-item scale as an indicator of clean sport behavior intentions.

#### 3.4.2 Structural model

**Figure 2:**
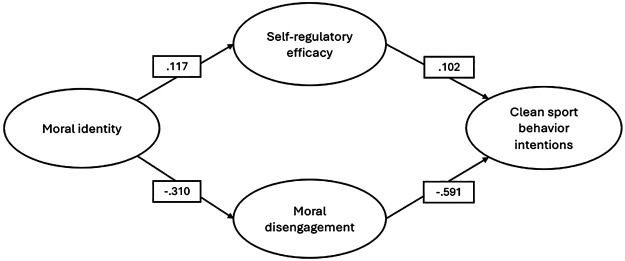
SEM path diagram. Results of the overall model.

Considering the hypothesized model, the SEM results showed a good fit to the data. Fit indices suggest the model adequately represented the observed data (CFI = .972; RMSEA = .048; SRMR = .057), meeting the commonly accepted criteria for an acceptable fit (45). Results of the overall model (Figure 2) showed a significant inverse relation between Moral identity and Moral disengagement (*β* = −.310, *p* < .0001) and on Self-regulatory efficacy (*β* = .011, *p* = .027). Finally, Clean sport behavior intentions are significantly predicted by Moral disengagement (*β* = −.591, *p* < .0001) and Self-regulatory efficacy (*β* = −.102, *p* = .03) (Figure 2). The pattern of indirect effects showed a significant indirect effect of Moral identity on clean sports behavior intentions mediated by Moral disengagement (*β* = .183, *p* < .0001).

Controlling for age improved the model fit (CFI = .972; RMSEA = .046; SRMR = .054), and age was significantly correlated with: Moral identity (*β* = .181, *p* < .001), Self-regulatory efficacy (*β* = .112, *p* < .05) and Clean sport behavior intentions *β* = .128, *p* < .01). Finally, adding age did not significantly alter the relationship between the variables.

A comprehensive multigroup analysis was performed to evaluate the structural model of both groups and to investigate statistically significant differences between French and Italian athletes (see Table 3). Multigroup comparisons revealed significant cross-national differences in the association between moral identity and moral disengagement (*β* = .171, *p* < .01), as well as in the relationship between self-regulatory efficacy and clean sport behavior intentions (*β* = .19, *p* < .01). No significant differences emerged for the effects of moral identity on self-regulatory efficacy or moral disengagement on clean sport behavior intentions. The fit indices and betas of both multigroup and non-multigroup models that include age have been added as Supplementary Material 3.

**Table 3:** Statistically significant differences in direct effects of multigroup analysis.

		French athletes		Italian athletes		
Dependent	Predictor	SE	*β*	SE	*β*	*P-Value*
Moral identity	Moral disengagement	.053	−.379	.027	−.208	.004
Moral identity	Self-regulatory efficacy	.175	.156	.082	.099	.768
Moral disengagement	Clean sport behavior intentions	.224	−.492	.464	−.582	.861
Self-regulatory efficacy	Clean sport behavior intentions	.032	.015	.051	.205	.001

Multigroup comparisons controlling for age revealed significant cross-national differences in the association between moral identity and moral disengagement (*β* = .171, *p* < .01), as well as in the relationship between self-regulatory efficacy and clean sport behavior intentions (*β* = .213, *p* < .001). These differences remained significant after controlling for age, indicating that they cannot be explained solely by age variation between the samples.

In both the Italian and French samples, the inclusion of age did not produce significant changes in any structural path, indicating that the model remained stable after controlling for age.

## 4 Discussion

The present study adds to anti-doping scholarship by examining the interplay of value-based and socio-cognitive influences on athletes' motivations to pursue clean sport practices. Importantly, behavior intentions are considered here as a component related to, but not identical with, clean sport identity itself. Departing from previous studies that have focused mainly on forecasting doping risk [e.g., (15, 27)], our approach centers on athletes' proactive engagement with ethical values. The evidence gathered highlights the significance of both moral identity and cognitive processes in shaping decisions to act ethically within sports settings. These outcomes extend the foundational work of Bandura (16), Ajzen (46), and Lucidi et al. (14), reinforcing the role of moral disengagement, self-regulatory efficacy, and moral identity in ethical conduct among athletes. According to the theoretical framework, moral identity shapes intentions to act ethically through its influence on self-regulatory efficacy and moral disengagement, offering new insights into the pathways by which moral factors affect clean sport intentions.

As a first step, we examined whether the measurement instruments functioned similarly for both French and Italian athletes. Building on this, we tested a model that identified moral identity as a key precursor to moral disengagement and self-regulatory efficacy, two factors thought to mediate its impact on intentions to maintain clean sport behaviors. In the final stage, we looked at whether this model held up consistently across both groups. The findings showed that the measurement model retained a stable structure and comparable factor loadings in each sample, which supports the reliability of comparing results between the groups (44, 47). Previous literature also supports the strong reliability and validity of these instruments in a range of countries (28, 31, 39, 42) and sport contexts (6, 14, 15).

The analysis using structural equation modeling (SEM) confirmed that the hypothesized model provided a satisfactory fit to the observed data. Of particular interest, the SEM results revealed a pronounced inverse relationship between moral identity and moral disengagement. This finding aligns with the notion that athletes who have a firmly rooted moral self-concept are less susceptible to the psychological mechanisms that facilitate disengagement (16, 20, 48). In practical terms, individuals with a strong moral identity often grapple more with ethical dilemmas, which in turn makes them less likely to justify or overlook rule violations (49, 50). Taken together, these findings highlight just how important moral identity is for fostering ethical choices in sports (51). What's more, the data show that athletes who see moral values as a core part of who they are tend to have greater confidence in their capacity to stick to those values, even when faced with challenging situations (16, 28). Within the model, moral disengagement emerged as the most influential predictor of intentions to engage in clean sport behavior. Earlier studies (14, 15) have similarly found that when athletes justify their actions to themselves, their moral barriers are lowered, making them more open to the idea of doping and less likely to resist it. Addressing this issue appears crucial, as research also shows that specific interventions aimed at reducing moral disengagement can actually decrease the likelihood of doping (42). The present study emphasizes the value of incorporating interventions that specifically target moral disengagement processes as part of prevention strategies. It also points to the need for a nuanced understanding of how moral disengagement and self-regulatory efficacy interact to shape athletes' intentions and actions (14, 15).

Self-regulatory efficacy emerged as a significant predictor of intentions to engage in clean sport behavior, but its effect was less pronounced than that of moral disengagement. This finding suggests that while confidence in resisting temptation is important, it must be anchored in a solid ethical foundation to consistently translate into intentions (17, 26). The model indicates that moral identity influences intentions to behave ethically mainly through its impact on moral disengagement rather than directly through self-efficacy. This distinction is noteworthy, as it shows that a strong moral self-concept is more effective in curbing harmful cognitive patterns than in merely boosting one's belief in their abilities. Practically, this implies that although self-efficacy is valuable, interventions should place greater emphasis on developing athletes' moral identity to more effectively shape behavior (32).

From a measurement standpoint, the clean sport behavioral intention scale, adapted from a pre-existing doping-likelihood measure, demonstrated robust factor loadings and good reliability, validating its structure. The scale's relationships with moral identity, moral disengagement, and self-regulatory efficacy also aligned with theoretical expectations, further supporting its validity within the present model [e.g., (36–38)].

The multigroup SEM analysis identified two statistically significant differences in the structural pathways between French and Italian athletes. First, the influence of moral identity on moral disengagement was notably stronger among French athletes. In contrast, the pathway from self-regulatory efficacy to clean sport behavior intentions was significantly stronger in Italian athletes. To speculate, while the first difference may be indicative of both cultural and developmental factors. With a higher average age, French athletes may possess a more developed moral self-concept, allowing moral identity to more effectively inhibit cognitive justifications for unethical behavior (21, 52). Variations in self-regulatory efficacy may be explained by the younger age of the Italian participants, whose beliefs about their ability to regulate their own behavior are still developing and thus more likely to influence their intentions. For athletes in the early stages of their careers, self-efficacy is particularly important in helping them withstand external pressures, especially when their moral identity is not yet fully formed (14, 26).

When age was included as a covariate in the analysis, it showed modest links to moral identity, self-regulatory efficacy, and intentions to engage in clean sport. This suggests that as athletes mature, they may go up developmental changes in both their sense of morality and their capacity for self-regulation. However, age did not have a significant relationship with moral disengagement, implying that these disengagement processes may be shaped more by situational, motivational, or cultural factors than by age itself.

Our findings show that socio-cognitive elements, especially moral disengagement, but also moral identity and self-regulatory efficacy, have a strong impact on athletes' intentions to participate in clean sport. This aligns with the growing body of research recognizing that behavioral intentions in sports are complex and shaped by multiple influences. For example, Bandura (16) noted that high self-regulatory efficacy can help athletes resist doping pressures and encourage ethical behavior. Adding to this, studies by Nicholls et al. (53) and Galli et al. (54) suggest that educational programs addressing both knowledge and self-regulation are effective in strengthening anti-doping intentions. Altogether, the evidence supports the idea that targeted interventions can positively influence athletes' choices regarding prohibited substances or methods. Based on our results, it seems clear that encouraging ethical intentions calls for a comprehensive approach, combining behavior change techniques, minimizing moral disengagement, supporting moral identity, and promoting positive attitudes about doping. Recent work (55, 56) further indicates that interventions focused on moral development can deepen athletes' commitment to clean sport. Educational strategies rooted in values aim to build integrity and improve ethical decision-making, particularly when athletes are confronted with dilemmas tied to performance. Ideally, such programs should be introduced early, so that ethical values become part of the foundation of an athlete's training (32).

Moreover, innovative research has advanced specific interventions using technologies, such as serious games, which enable athletes to simulate contexts and real-life scenarios. These tools allow them to confront moral dilemmas and build greater confidence in their capacity to act ethically (4, 54). Given this evidence, our study is part of a broader project to develop innovative preventive interventions utilizing digital technologies, such as serious games, based on socio-cognitive dimensions to promote clean sport behavior intentions in a cross-national context (Italy and France).

### 4.1 Strengths and limitations

This study utilizes models previously analyzed in doping research [e.g., (14, 16, 45, 57)], refining the relationship between socio-cognitive variables and the moral aspects of behavior. In particular, it incorporates self-regulatory efficacy and moral disengagement as key mediators between the moral identity and the clean sport behavior intentions. Integrating these variables enriches the theoretical model and clarifies the complex pathways that shape athletes' decision-making in ethically challenging contexts. Another strength of this study is its cross-national approach. The comparison between Italy and France underscores the need to consider national differences when analyzing the relationships between socio-cognitive variables through an invariance analysis. This approach allowed us to identify similarities and differences in the socio-cognitive mechanisms operating in each country. The multigroup analysis revealed general consistencies across the two samples. However, we found that self-efficacy had a weaker effect on intentions among French athletes than Italian athletes. These differences may reflect national variations in perceived personal control and social norms related to doping, as previously suggested by Woolway and colleagues (31).

This research forms one component of a broader longitudinal project, using the collected data to create prevention materials for amateur athletes in track and field. At this stage, we have not yet implemented digital platforms like serious games or e-learning in our interventions. However, recent literature [e.g., (4, 54)] points to the value of these tools in altering socio-cognitive factors that support long-term commitment to clean sport. Their flexibility means such platforms can be adapted for different sports and countries, potentially broadening their impact on anti-doping efforts.

Our findings point to the importance of including both moral and socio-cognitive elements in models that seek to predict doping behavior, echoing theories that stress the central role of moral disengagement. Because moral identity and national context both matter, it makes sense to tailor anti-doping programs to the needs of particular groups and to build in educational components that help athletes develop strong ethical values and identities. Still, as Black et al. (57) note, more work is required to fully understand how moral disengagement operates as a mediator, especially in high-pressure competitive settings where its influence might be heightened.

Furthermore, the study did not account for years of sport participation, which may represent an important developmental factor influencing moral identity and regulatory processes. Future studies should examine the dynamic interactions between moral identity and self-regulatory efficacy across different levels of competition, using longitudinal approaches to refine and further validate the predictive model.

Additionally, future research should investigate whether these relationships operate similarly across different sport contexts, as moral climates and regulatory demands may vary substantially between sport types.

### 4.2 Conclusions

Our study significantly contributes to doping research, highlighting the important role of moral factors in shaping athletes' intentions to promote clean sport behavior. The cross-national comparison between Italy and France offers a deeper understanding of the different contexts that influence athletes' decision-making processes.

An innovative aspect of this study is its relevance for the development of interventions to promote clean behavior. Specifically, our findings provide a solid theoretical foundation for the creation of targeted educational strategies and innovative digital tools, such as serious games, focused on moral considerations. These tools could foster lasting change in athletes' intentions to remain clean. Furthermore, the adaptability of these platforms to different cultural contexts increases their effectiveness in prevention strategies, making them promising resources for future interventions in different sports and national contexts.

## Data Availability

The data that support the findings of this study are openly available in “WADA_Animate” at https://osf.io/ahzqj/overview
