# Correction: The effects of athletes’ moral identity on clean sport behavior intentions: a cross-national study of the mediating role of moral disengagement and self-efficacy

**DOI:** 10.3389/fspor.2026.1840128

**Published:** 2026-04-07

**Authors:** Martina Giorgi, Andrea Chirico, Tommaso Palombi, Vindice Deplano, Daniel Gotti, Alessandra De Maria, Federica Galli, Luca Mallia, Roberto Codella, Arnaldo Zelli, Fabio Lucidi

**Affiliations:** 1 Department of Psychology of Social and Developmental Processes, Sapienza University of Rome, Rome, Italy; 2 Department of Biomedical Sciences for Health, University of Milan, Milan, Italy; 3 Department of Movement, Human and Health Sciences, University of Rome “Foro Italico”, Rome, Italy

**Keywords:** clean sport behavior intentions, cross-national study, doping prevention, moral disengagement, moral identity, quantitative study, self-regulatory efficacy, socio-cognitive model

Figures 1 and 2 were in the wrong order. The captions and the citations within the text were correct, but they referred to the wrong images. The order has now been corrected.

The original version of this article has been updated.

